# Impact of contrast ultrasound diagnosis for patients with liver cancer

**DOI:** 10.1097/MD.0000000000015445

**Published:** 2019-05-13

**Authors:** Hong-bin Guo, Jun-hu Wang

**Affiliations:** a Department of Ultrasound, Second Affiliated Hospital of Xi’an Medical College, Xi’an; b Department of Ultrasound Diagnosis, Yan’an People's Hospital, Yan’an, China.

**Keywords:** contrast ultrasound, liver cancer, sensitivity, specificity

## Abstract

**Background:**

Numerous studies have reported that contrast ultrasound (CU) can be utilized for diagnosis in patients with liver cancer (LC) accurately. However, no systematic review has addressed to assess its diagnostic impact on patients with LC. Thus, this systematic review will investigate the accurate of CU diagnosis on LC.

**Methods:**

A comprehensive literature search for relevant studies will be performed in the Cochrane Library, EMBASE, MEDILINE, Web of Science, PSYCINFO, Cumulative Index to Nursing and Allied Health Literature, Allied and Complementary Medicine Database, Chinese Biomedical Literature Database, and China National Knowledge Infrastructure from inceptions to the March 10, 2019. All case-controlled studies investigating the impacts of CU diagnosis on LC will be included in this study. Two researchers will independently carry out study selection, quality assessment, and data extraction. The quality will be assessed by using Quality Assessment of Diagnostic Accuracy Studies tool. Statistical analysis will be conducted by RevMan V.5.3 (Cochrane Community, London, UK) and Stata V.12.0 software (Stata Corp, College Station).

**Results:**

This study will present the accuracy of CU diagnosis for patients with LC through the assessment of sensitivity, specificity, positive likelihood ratio, negative likelihood ratio, and diagnostic odds ratio of CU.

**Conclusion:**

The findings of this study will summarize the current evidence for accuracy of CU diagnosis in patients with LC.

**Systematic review registration:**

PROSPERO CRD42019127108.

## 1 Introduction

Liver cancer (LC) is one of the most common digestive system malignant tumors.^[1,2]^ Many factors can cause LC, such as chronic infection with hepatitis B virus or hepatitis C virus, cirrhosis, certain inherited liver diseases, diabetes, nonalcoholic fatty liver disease, exposure to aflatoxins, and excessive alcohol consumption.^[3–7]^ Its mortality rate is relatively high, because it is asymptomatic at early stage and not easy to be identified.^[8,9]^ Most patients are identified as LC with symptoms manifestation until it has advanced to the late stage, which seriously impacts the management and prognosis of the patients.^[8,9]^ Thus, it is very important to diagnosis and to treat it at early stage.^[10]^

Lots of previous studies have reported that contrast ultrasound (CU) has been used to diagnosis patients with LC.^[11–20]^ However, no study has been conducted to assess the accurate of CU diagnosis on LC. Thus, this study will firstly explore the accurate diagnosis of CU for patients with LC.

## 2 Methods

### 2.1 Objective

This study will aim to systematically investigate the value of CU in the diagnosis of patients with LC.

### 2.2 Ethics and dissemination

This study will not inquire ethic approval, because we will not analyze individual patient data. Its findings will be published on peer-reviewed journals.

### 2.3 Study registration

This study has been registered on PROSPERO CRD42019127108. We have reported it according to the guideline of preferred reporting items for systematic reviews and meta-analysis protocol (PRISMA-P) statement.^[21]^

### 2.4 Eligibility criteria for study selection

#### 2.4.1 Type of studies

Case-controlled studies on the diagnostic accuracy of CU for patients with LC will be included in this study. However, non-clinical researches, non-controlled trials will not be considered for inclusion.

#### 2.4.2 Type of participants

Any patients with histological-proven LC will be included in this study, regardless their race, age, sex, and economic status.

#### 2.4.3 Type of index test

Index test will include CU for the diagnosis of LC. We will exclude the combined diagnosis of CU with other test.

Reference test: patients with histological-proven LC will be included in the control group.

#### 2.4.4 Type of outcome measurements

Primary outcomes comprise of sensitivity and specificity. Secondary outcomes include positive likelihood ratio, negative likelihood ratio, and diagnostic odds ratio.

### 2.5 Data sources and search strategy

We will performance a comprehensive literature search for relevant studies from Cochrane Library, EMBASE, MEDILINE, Web of Science, PSYCINFO, Cumulative Index to Nursing and Allied Health Literature, Allied and Complementary Medicine Database, Chinese Biomedical Literature Database, and China National Knowledge Infrastructure from inceptions to the March 10, 2019. All case-controlled studies exploring the impacts of CU diagnosis on LC will be included in this study. In addition, we will also search reference lists of all eligible studies, and relevant reviews. Search strategy for Cochrane Library is demonstrated in Table 1. We will also use identical search strategies for other electronic databases.

**Table 1:**
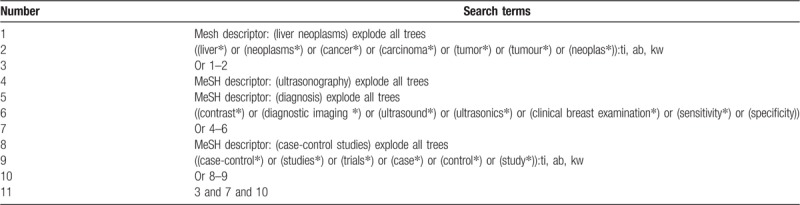
Search strategy used in Cochrane Library database.

### 2.6 Data collection and management

#### 2.6.1 Study selection

All searched records will be imported into Endnote 7.0 software (Clarivate Analytics, Philadelphia), and duplication studies will be removed. Two researchers will independently scan the titles and abstracts for the remaining records to exclude any irreverent studies. Then, they will read full texts to further determine if those studies can meet the final inclusion criteria. Any disagreements will be solved by a third researcher through discussion. The results of study selection will be summarized according to the PRISMA flow diagram.

#### 2.6.2 Data collection

Two researchers will collect important information and extract data from all eligible studies independently by using predefined data collection form. A third researcher will help to resolve any divergences between 2 researchers through discussion. We will collect following information:

(1) Study general information: title, authors, year of publication, region, etc;
(2) Patient general information: race, age, sex, diagnostic criteria, etc;
(3) Diagnostic details: index test and any reference tests;
(4) Study methods: sample size, randomization, blinding, etc;
(5) Outcome measurements: number of true positives and negatives, false positives and negatives for each diagnostic test, etc.

#### 2.6.3 Managing missing data

We will contact original authors by email if there is any missing data, or insufficient information. We will conduct data synthesis through available data if the missing data are not available.

### 2.7 Quality assessment for eligible studies

We will utilize Quality Assessment of Diagnostic Accuracy Studies (QUADAS-2) tool to assess quality for all eligible studies on 4 aspects.^[22]^ Each aspect is assessed with risk of bias, measuring by signaling questions. Two researchers will investigate the quality for each eligible study independently. Any conflicts regarding the quality assessment between 2 researchers will be resolved by discussion with another researcher.

### 2.8 Statistical analysis

This study will utilize RevMan V.5.3 and Stata V.12.0 software to perform statistical analysis. Stata V.12.0 software will be used to plot estimates of sensitivity, specificity, positive likelihood ratio, negative likelihood ratio, and diagnostic odds ratio. Heterogeneity among eligible studies will be identified by *I*^2^ statistic. *I*^2^ ≤50% suggests low heterogeneity, and a fixed-effect model will be used. Otherwise, *I*^2^ >50% suggests significant heterogeneity, and a random-effect model will be used. In addition, subgroup analysis will be performed to explore any potential causes. Under such situation, bivariate random-effect regression approach will be used for estimating of sensitivity and specificity.

Subgroup analysis will be performed to determine the possible reasons that may cause significant heterogeneity according to the different types of LC, study, and patient characteristics. In addition, we will also carry out sensitivity analysis by eliminating the low quality studies. Furthermore, funnel plots and Egger linear regression test will be performed to identify any feasible reporting bias if >10 eligible studies are included.^[23]^

## 3 Discussion

This study will firstly explore the diagnostic accuracy of CU in patients with LC through evaluating its sensitivity, specificity, positive likelihood ratio, negative likelihood ratio, and diagnostic odds ratio. The results of this study will provide a summary of the updated evidence on the diagnostic accuracy of CU for LC, which is helpful for diagnosis and treatment of LC at early stage.

## Author contributions

**Conceptualization:** Hong-bin Guo, Jun-hu Wang.

**Data curation:** Hong-bin Guo, Jun-hu Wang.

**Formal analysis:** Hong-bin Guo.

**Funding acquisition:** Jun-hu Wang.

**Investigation:** Jun-hu Wang.

**Methodology:** Hong-bin Guo.

**Project administration:** Jun-hu Wang.

**Resources:** Hong-bin Guo.

**Software:** Hong-bin Guo.

**Supervision:** Jun-hu Wang.

**Validation:** Hong-bin Guo, Jun-hu Wang.

**Visualization:** Hong-bin Guo, Jun-hu Wang.

**Writing – original draft:** Hong-bin Guo, Jun-hu Wang.

**Writing – review & editing:** Hong-bin Guo, Jun-hu Wang.
